# Supplementary material for: Structure and functional dynamics of the mitochondrial Fe/S cluster synthesis complex
Source: Nat Commun. 2017 Nov 3;8:1287. doi: 10.1038/s41467-017-01497-1 (PMC5668364; doi:10.1038/s41467-017-01497-1)
Supplement: Supplementary file 1 — Supplementary Information [file 41467_2017_1497_MOESM1_ESM.pdf]

Supplementary Figure 1

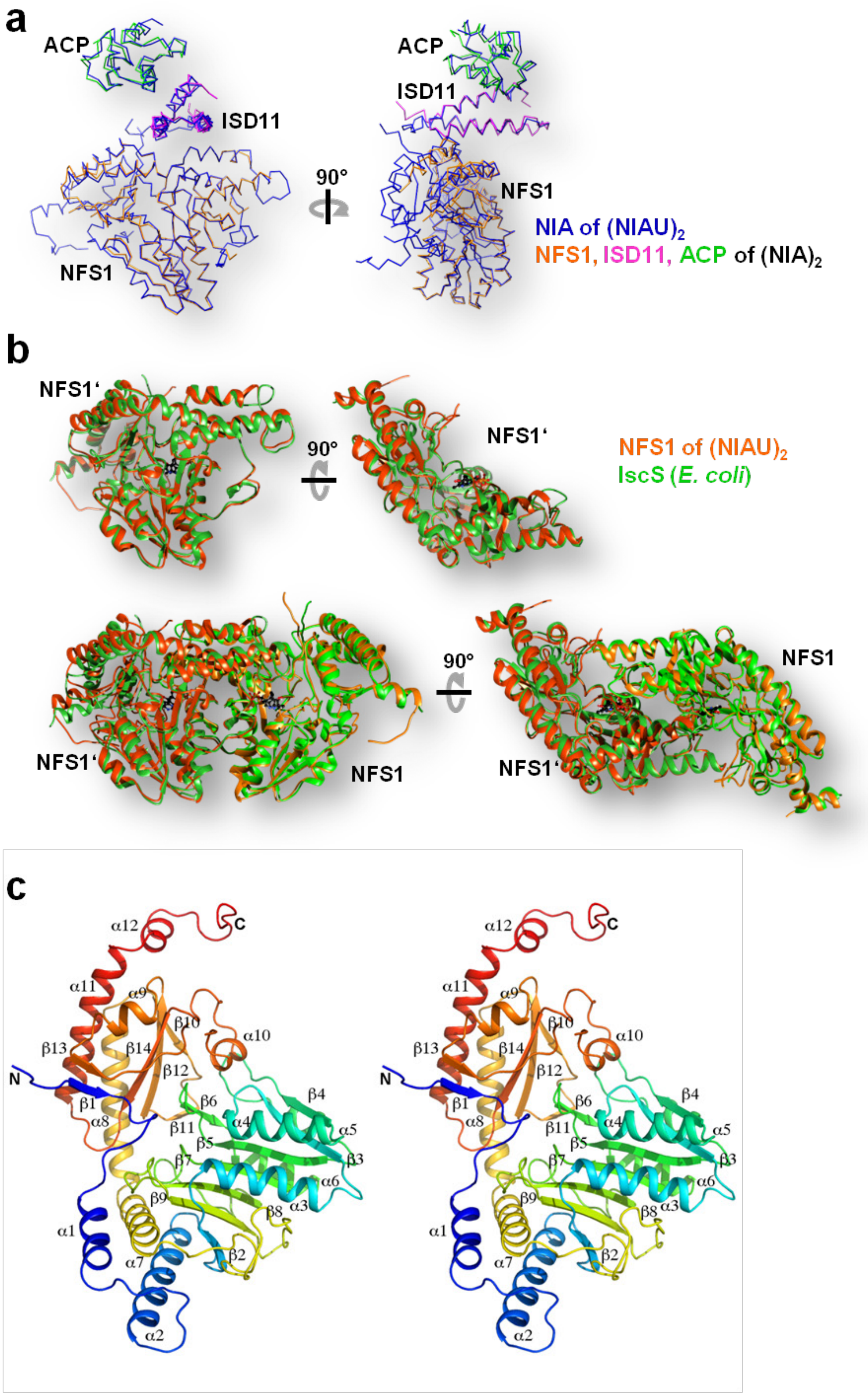

d

```
        10         20         30         40         50
MLLRAAWRRA AVAVTAAPGP KPAAPTRGLR LRVGDRAPQS AVPADTAAAP
        β1               α1                     α2
        60         70         80         90        100
EVGPVLRPLY MDVQATTPLD PRVLDAMLPY LINYYGNPHS RTHAYGWESE
                        β2          α3          β3
       110        120        130        140        150
AAMERARQQV ASLIGADPRE IIFTSGATES NNIAIKGVAR FYRSRKKHLI
                α4        β4              α5        β5
       160        170        180        190        200
TTQTEHKCVL DSCRSLEAEG FQVTYLPVQK SGIIDLKELE AAIQPDTSLV
           β6        α6        β7
       210        220        230        240        250
SVMTVNNEIG VKQPIAEIGR ICSSRKVYFH TDAAQAVGKI PLDVNDMKID
  β8             β9
       260        270        280        290        300
LMSISGHKIY GPKGVGAIYI RRRPRVRVEA LQSGGGQERG MRSGTVPTPL
    α7                α8                 β10      β11
       310        320        330        340        350
VVGLGAACEV AQQEMEYDHK RISKLSERLI QNIMKSLPDV VMNGDPKHHY
    β12        α9        β13               α10
       360        370        380        390        400
PGCINLSFAY VEGESLLMAL KDVALSSGSA CTSASLEPSY VLRAIGTDED
    β14                  α11               α12
       410        420        430        440        450
LAHSSIRFGI GRFTTEEEVD YTVEKCIQHV KRLREMSPLW EMVQDGIDLK

SIKWTQH
```

α-helices – **red,** short helical turns – **green,** β-sheet – **blue.**
The construct starts at residues 56, residues 1-55 – **gray.**
The C-terminus is visible to a different degree in various NFS1 copies. His457 (**gray**) is disordered in all molecules.
Underlined residues are disordered only in the NIA complex.
Residues underlined with a dashed line (381-385) are disordered in the NIAU complex.
All residues are visible in the electron density map of the NIAU-Zn complex.

Supplementary Figure 1 | Comparison of 3D structures observed in different complexes. **a**, Superposition of the NFS1-ISD11-ACP sub-complex of the $(NIA)_2$ and $(NIAU)_2$ complexes shown as backbone chain trace. The backbone of the $(NIAU)_2$ sub-complex is shown in blue, that of $(NIA)_2$ in orange, magenta and green for NFS1, ISD11 and ACP, respectively. The arrangement of NFS1, ISD11, and ACP are virtually identical in all complexes. **b**, Superposition of the crystal structures of NFS1 part of the $(NIAU)_2$ complex (orange) and *E. coli* IscS (green; PDB code 3LVL) [1] (top: monomer, bottom: dimer). **c**, Stereo view of human NFS1 taken from the $(NIAU)_2$-Zn complex with secondary structure assignments in rainbow coloring (N terminus in blue to C terminus in red). **d**, Primary structure of human NFS1 with secondary structure assignments.

Supplementary Figure 2

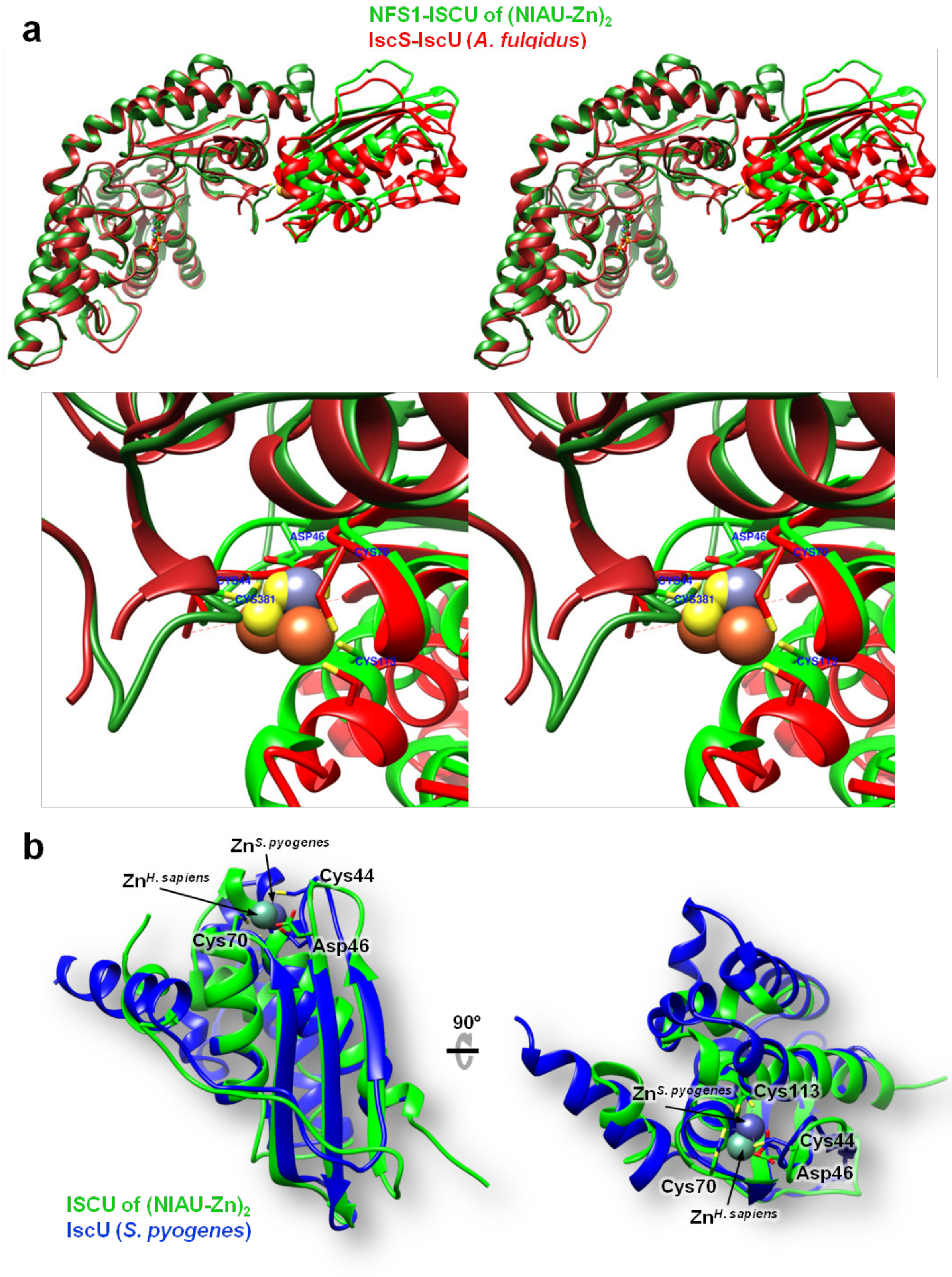

c

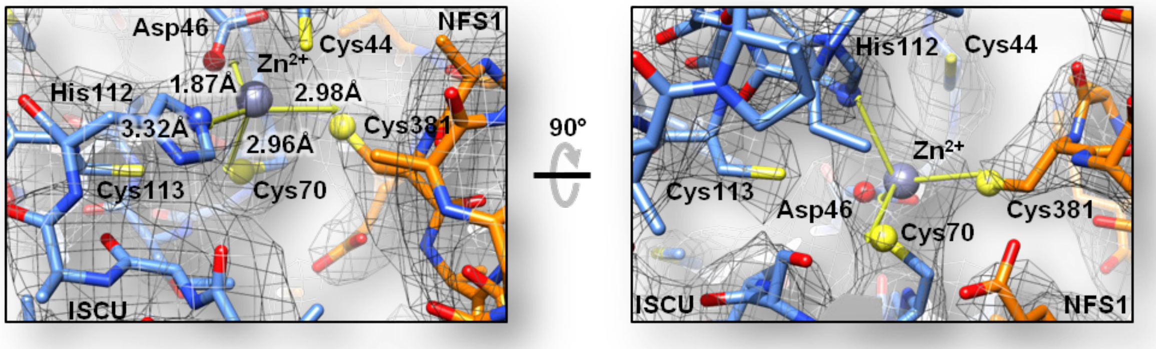

Supplementary Figure 2 | Coordination geometry of Zn between NFS1 and ISCU. **a**, Stereo view of a superposition of the human NFS1-ISCU sub-complex (green, taken from the $(NIAU\text{-}Zn)_2$ complex) with IscS-IscU from *A. fulgidus* (red, PDB code 4EB7)[2]. The bottom panel shows a stereo view of the Zn or Fe/S cluster binding regions of NFS1-ISCU or IscS-IscU complexes, respectively. Zn is shown in slate grey, the Fe/S cluster in red-brown and yellow. Zn-coordinating residues are labeled in blue for NFS1-ISCU. **b**, Superposition of human ISCU taken from the $(NIAU\text{-}Zn)_2$ complex (green) and *Streptococcus pyogenes* IscU (blue; PDB code: 1SU0), both in complex with Zn (depicted as spheres in the respective pale color) [3]. Residue numbering refers to human NFS1. **c**, Detailed view of the Zn coordination in the $(NIAU\text{-}Zn)_2$ complex within the crystal-derived electron density map. ISCU is depicted in blue and NFS1 is shown in orange. Zn (dark slate blue) is coordinated in a tetrahedral fashion. Yellow sticks represent an idealized tetrahedral geometry. Coordinating atoms of Cys381^{NFS1} and Asp46, Cys70, and His112 of ISCU are shown as balls and bond distances are given. Cys44 and Cys113 of ISCU are not involved in Zn coordination.

Supplementary Figure 3

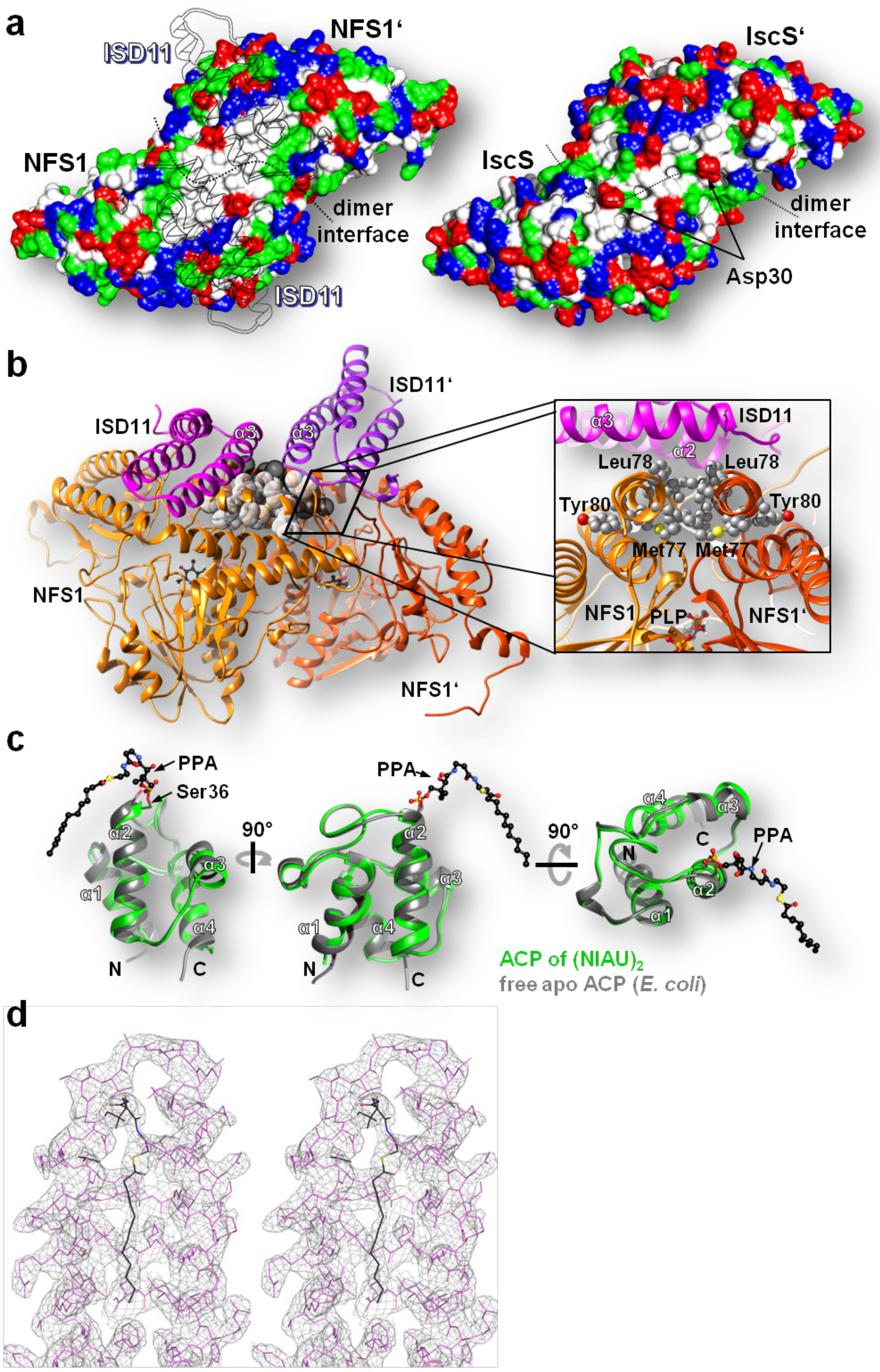

Supplementary Figure 3 | ISD11 and ACP are bound distant from the NFS1 catalytic site in the ISC complexes. a, Comparison of the surfaces of NFS1 and *E. coli* IscS (PDB code 3LVL). Model for NFS1 was taken from the $(NIAU)_2$ complex. Hydrophobic (white), polar (green), positively (blue) and negatively (red) charged residues and the dimer interfaces are highlighted. The binding region of the ISD11 dimer is depicted as silhouette on top of NFS1. **b**, The hydrophobic patch on the top of NFS1 (part **a**) is part of the NFS1-ISD11 interface. Hydrophobic residues (grey) are represented as balls, NFS1 (orange) and ISD11 (magenta) as ribbon. The inset shows NFS1 residues involved in interaction with ISD11 (for clarity only one ISD11 molecule with labeled helices is shown). **c**, Superposition of ACP (green) from the $(NIAU)_2$ complex and free apo-ACP (grey, PDB code 2FAE [4]). The phosphopantetheine (PPA) attached to Ser36 and the fatty acyl chain are shown as sticks. **d**, ACP-linked PPA and its fatty acyl chain (black) inside the crystal-derived electron density within the hydrophobic tunnel of ISD11 (magenta).

Supplementary Figure 4

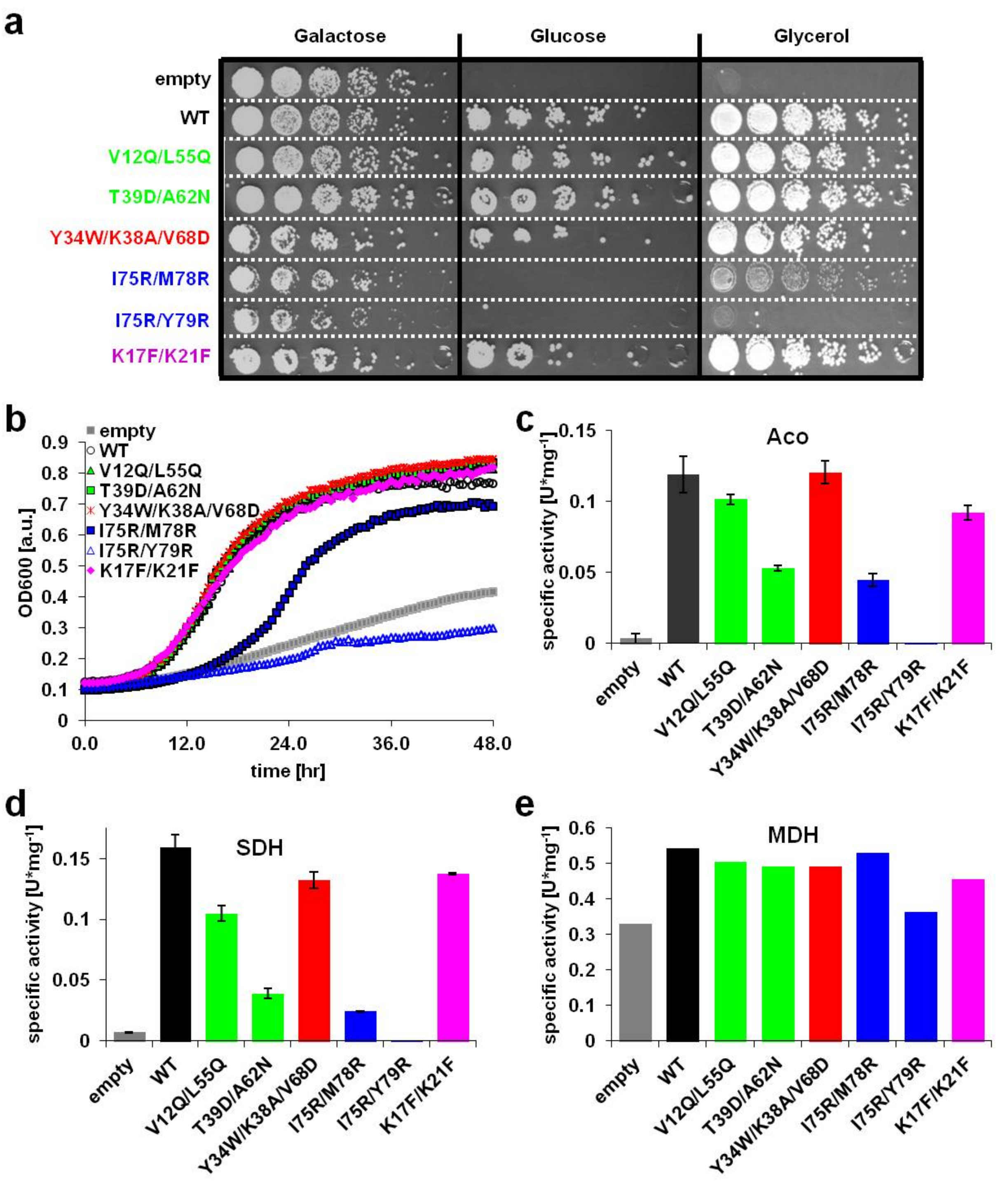

Supplementary Figure 4 | Analyses of *ISD11* mutants in yeast. a, Gal-ISD11 yeast cells were transformed with plasmids encoding either no protein (empty) or wild-type (WT) or the indicated FLAG-tagged Isd11 mutant proteins (Fig. 4a). Gal-ISD11 yeast cells were depleted of endogenous Isd11 on glucose medium for 40 h. Fivefold serial dilutions of cells were spotted on agar plates with the indicated carbon sources to screen for growth defects. **b**, Cells from above were analyzed for growth defects in liquid minimal medium supplemented with glucose. **c-e**, The specific enzyme activities ($U*mg^{-1}$) of aconitase (**c**), SDH (**d**) and MDH (**e**) were measured in Gal-ISD11 cells producing the indicated Isd11 mutant proteins (raw data for Fig. 4e-f). The error bars indicate the SD (n≥3).

Supplementary Figure 5

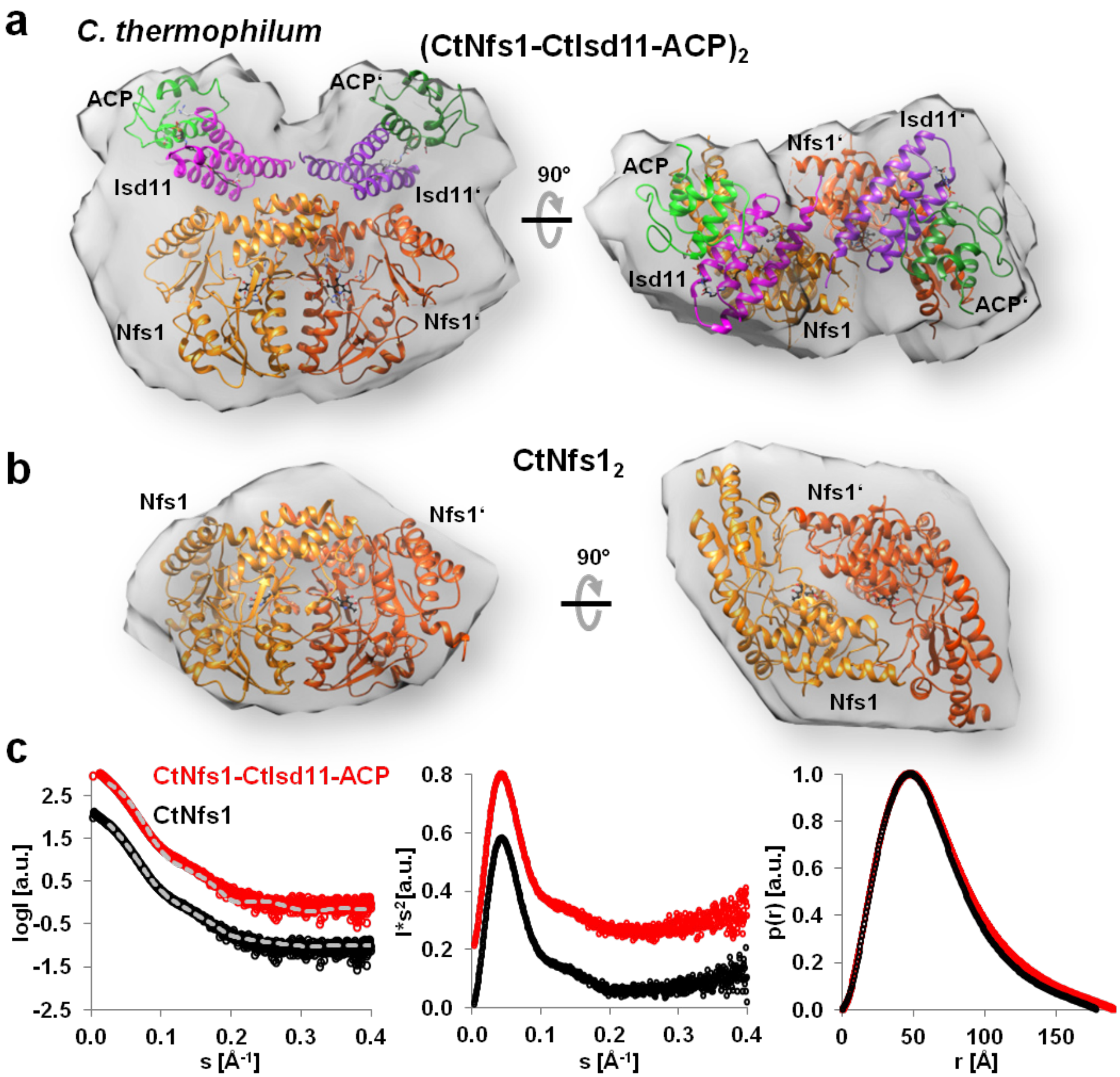

d

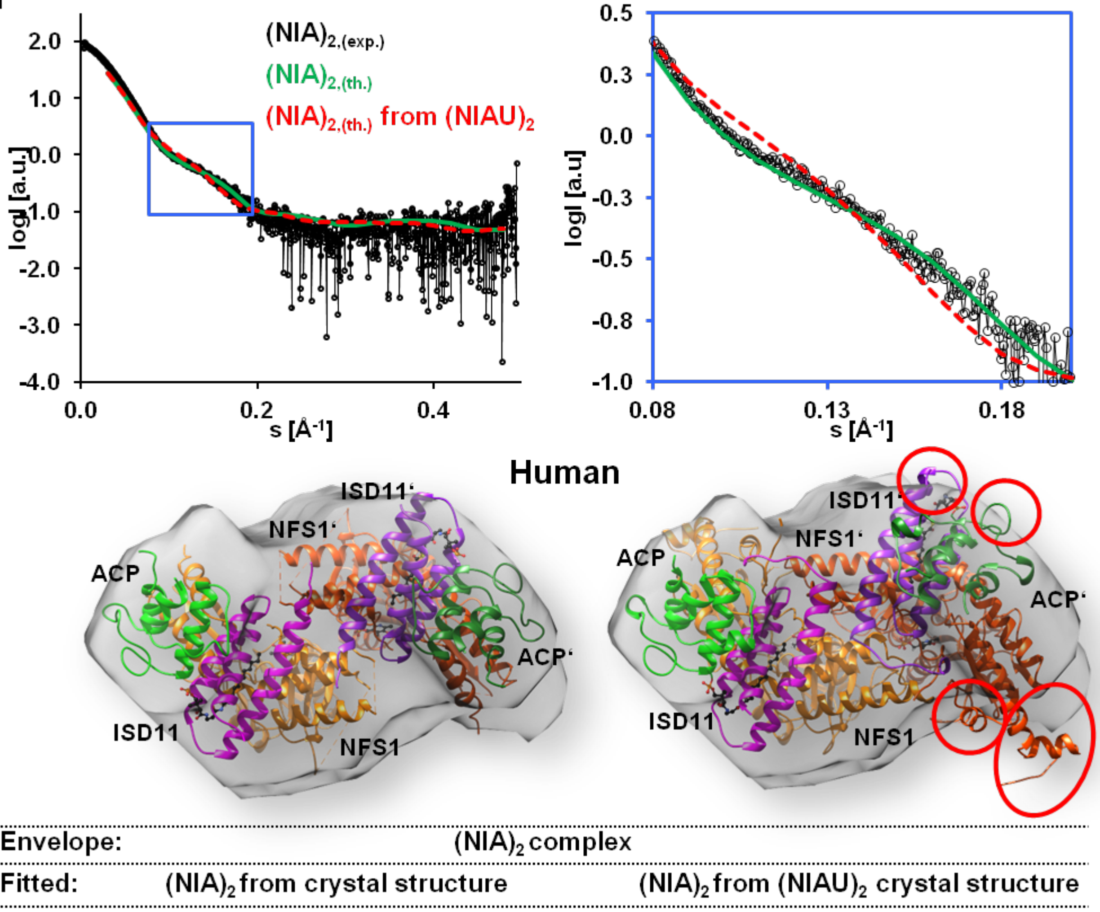
(NIA)2,(exp.)
(NIA)2,(th.)
(NIA)2,(th.) from (NIAU)2
logI [a.u.]
s [Å-1]
Human
ISD11'
NFS1'
ACP
ACP'
ISD11
NFS1
Envelope: (NIA)2 complex
Fitted: (NIA)2 from crystal structure
(NIA)2 from (NIAU)2 crystal structure

e

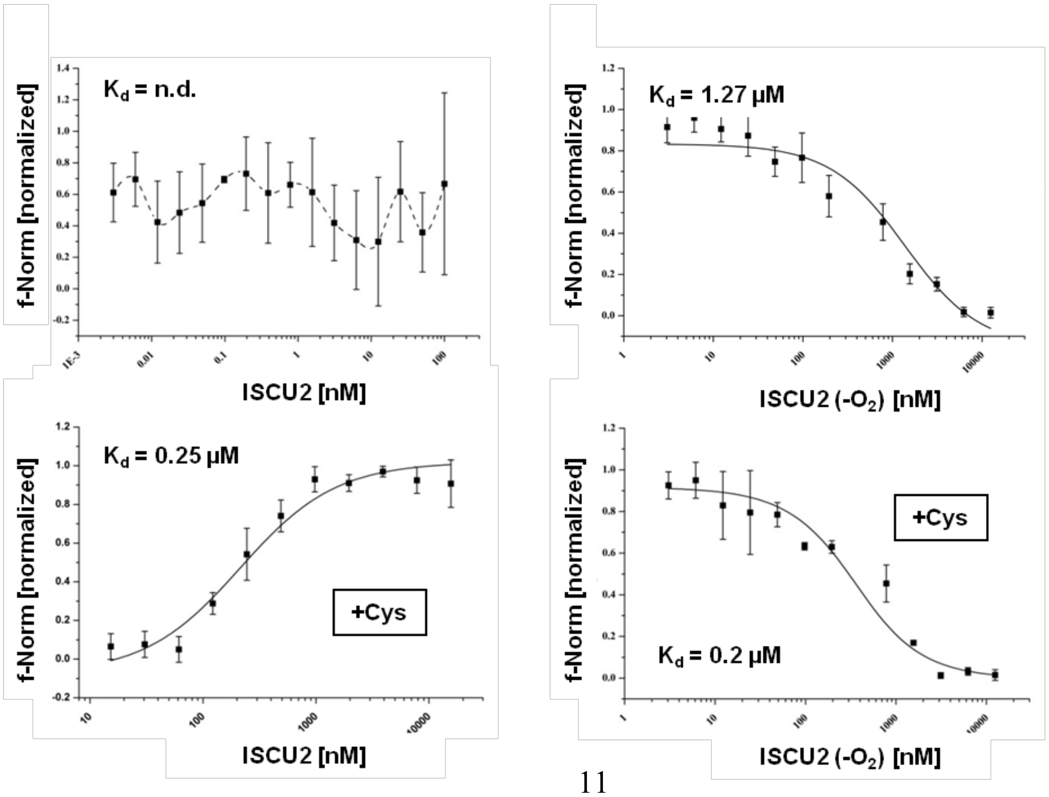
Kd = n.d.
Kd = 1.27 µM
Kd = 0.25 µM
Kd = 0.2 µM
+Cys
+Cys
f-Norm [normalized]
ISCU2 [nM]
ISCU2 (-O2) [nM]

Supplementary Figure 5 | SAXS shapes of *C. thermophilum* CtNfs1 with and without bound CtIsd11-ACP. a-b, SAXS analyses were performed for CtNfs1 with (**a**) and without (**b**) bound CtIsd11-ACP. Crystal structures of the human (NIA)2 complex (**a**) or of the NFS1 dimer part alone (**b**) were fit into the determined SAXS densities. **c**, Scattering curves with respective fits in grey (left) and Kratky plots (middle) of $(NIA)_2$ (red) and $NFS1_2$ (black) show no signs of aggregation and suggest a homogeneous solution of the respective complex. The pair distribution plots (right) show a bigger D_{max} for $(NIA)_2$ compared to $NFS1_2$ alone. **d**, Fitting of the calculated scattering amplitudes of the human $(NIA)_2$ complex taken from either $(NIA)_2$ (green) or $(NIAU)_2$ (red) to the experimental SAXS data of the $(NIA)_2$ complex (top left). The theoretical scattering curve of $(NIA)_2$ taken from $(NIAU)_2$ does not fit the experimental data well (top right). The bottom panel illustrates the fitting of the two complex parts into the SAXS envelope of $(NIA)_2$. The $(NIA)_2$ sub-complex from $(NIAU)_2$ does not fit well into the SAXS-derived density. Red circles mark the regions of poor or no fit. **e,** Raw data of the equilibrium titrations of CtNFS1 with CtIsu1 by microscale thermophoresis. K_d values are shown as insets. CtNfs1 was titrated with the amount of the respective protein shown in the plot. The error bars indicate the SD of three biological replicates each including three technical replicates.

Supplementary Figure 6

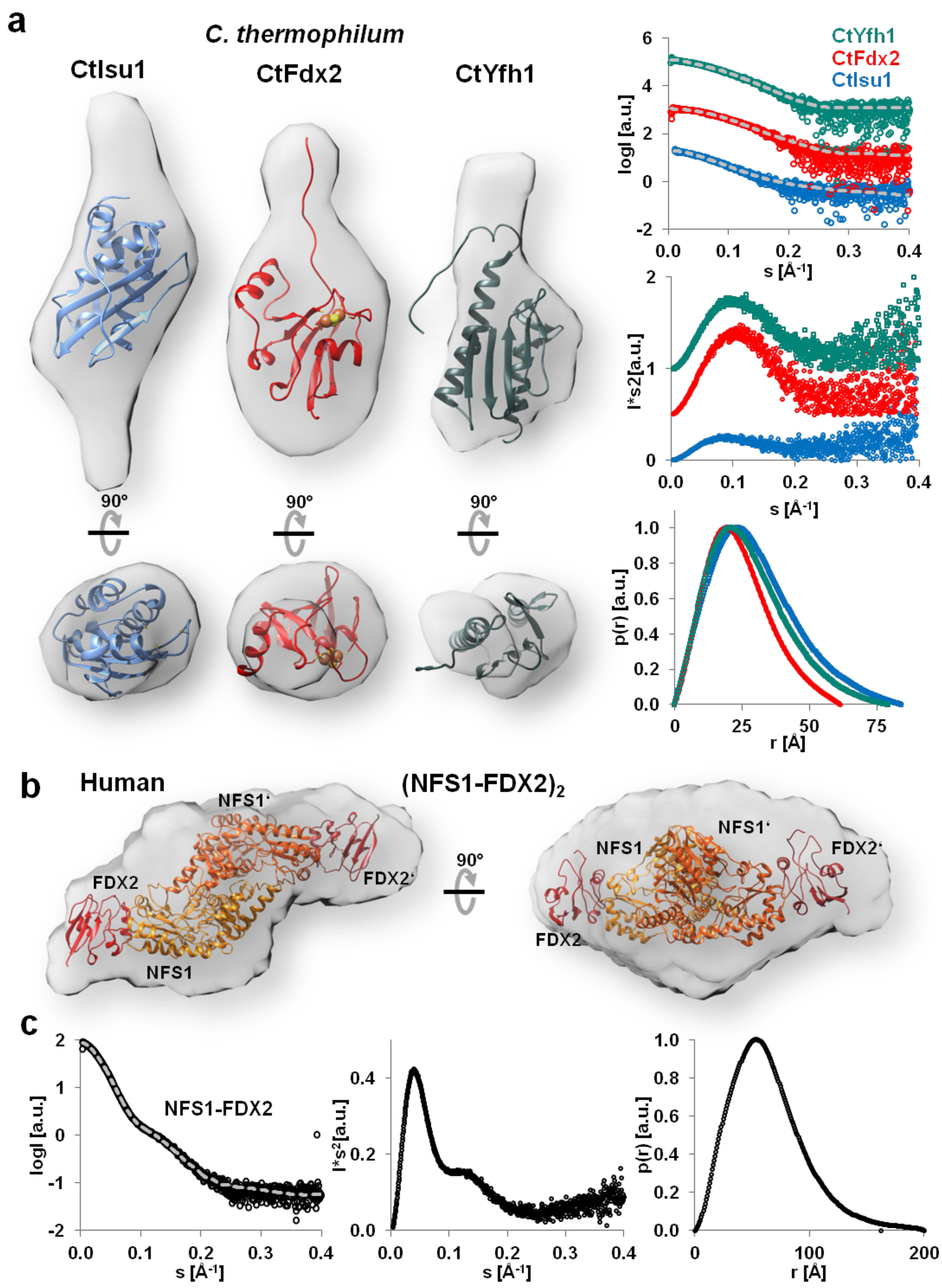

Supplementary Figure 6 | SAXS shapes of *C. thermophilum* ISC proteins and of human (NFS1-FDX2)$_2$ complex. a, SAXS results for the respective monomeric subunits from *C. thermophilum*. ISCU (blue) taken from the (NIAU)$_2$ complex was fit into the SAXS density. For ferredoxin (red) the NMR solution structure of Yah1 (PDB code 2MJE) was fit. In case of frataxin (dark slate grey) the yeast structure (PDB code 2GA5) was fit into the SAXS derived density. Scattering curves with respective fits (grey dashed lines in right top panel) and Kratky plots (right, middle) are consistent with homogeneous monomeric proteins in solution. The pair distribution plots (right, bottom) show the respective D_{max} values for monomeric proteins. **b**, Human NFS1 (orange) was incubated with human FDX2 (red) in a 1:1 ratio. NFS1 and FDX2 were aligned to the complex obtained from *C. thermophilum* proteins (Fig. 6b) and the resulting model was fit to the determined SAXS density. **c**, Scattering curve with respective fits (grey dashed lines in left panel), Kratky plot (middle), and pair distribution plot (right) for part **b**.

Supplementary Figure 7

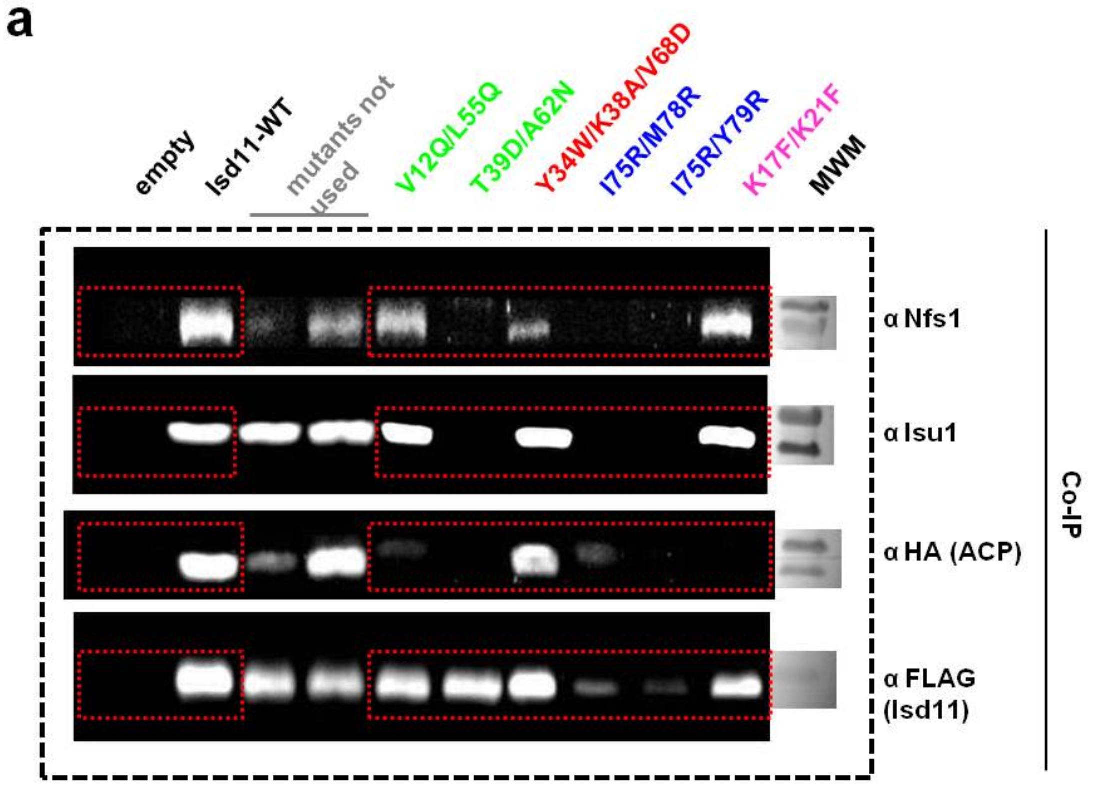

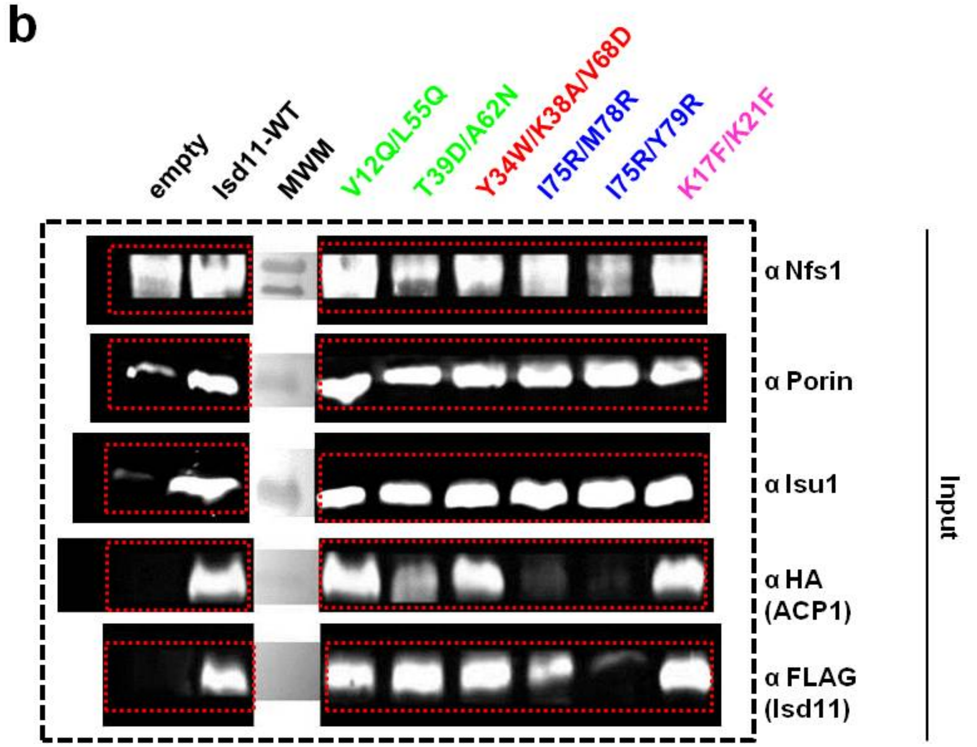

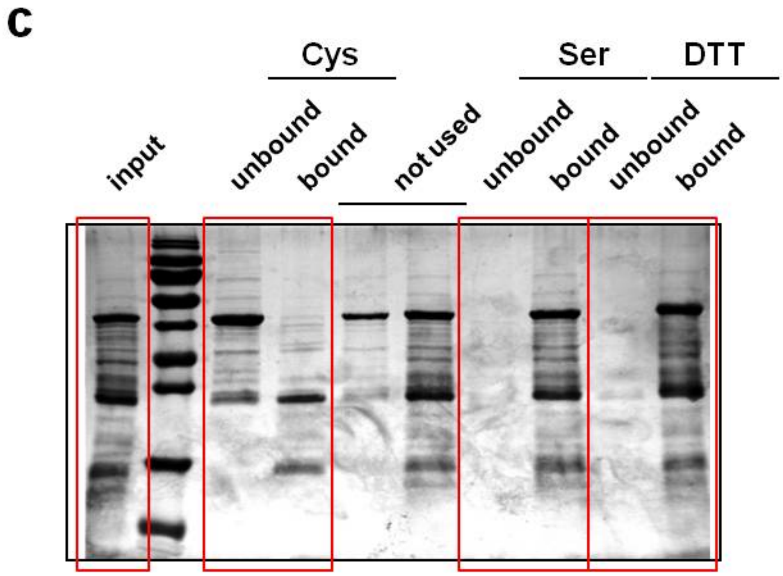

Supplementary Figure 7 | Images of uncropped blots and gels. a-b, Blots from Fig. 4b. Nitrocellulose membrane was cut according to the size of the respective protein and immunostained by the respective antibody. For antibody specifications and dilutions refer to Methods section. “mutants not used”: Two additional *ISD11* mutants were generated but not used in this study. **b**, Uncropped Coomassie-stained SDS polyacrylamide gel from Fig. 5e. “not used”: Data was not used for this study.

Supplementary Table 1. SAXS statistics (*C. thermophilum*).

	Radius of gyration R_g (Å)	Molecular mass MM_{ex} (kDa)	Molecular mass MM_{th} (kDa)	Best Chi value (χ^2)
CtIsu1	22.5 +/- 2.4	17.0	15.6	1.58
CtFdx2	18.5 +/- 2.3	11.3	13.1	0.82
CtYfh1	20.5 +/- 4.0	16.0	17.0	1.77
$CtNfs1_2$	41.6 +/- 0.1	92.9	91.8	7.42
$Ct(Nfs1\text{-}Isd11\text{-}ACP)_2$	51.5 +/- 0.1	127.3	137.1	2.71
$Ct(Nfs1\text{-}Isu1)_2$	33.4 +/- 0.2	121.6	123.1	11.72
$Ct(Nfs1\text{-}Fdx2)_2$	34.0+/- 0.0	115.5	118.0	2.57
$Ct(Nfs1\text{- }Isu1\text{-}Fdx2)_2$	37.2 +/- 0.4	148.1	149.3	2.89
$Ct(Nfs1\text{- }Isu1\text{-}Yfh1)_2$	40.6 +/- 0.2	167.5	157.0	9.75
$Ct(Nfs1\text{-}Fdx2\text{-}Yfh1)_2$	36.3 +/- 1.5	151.6	151.9	10.78
$Ct(Nfs1\text{-}Isu1\text{-}Fdx2\text{-}Yfh1)_2$	38.5 +/- 1.1	176.5	183.2	8.46

Supplementary Table 2. SAXS statistics *(H. sapiens)*

	Radius of gyration R_g (Å)	Molecular mass MM_{ex} (kDa)	Molecular mass MM_{th} (kDa)	Best Chi value (χ^2)
$NFS1_2$	47.4 +/- 0.0	92.0	100.6	4.62
$(NFS1\text{-}ISD11\text{-}ACP)_2$	54.7 +/- 0.2	136.6	146.7	0.95
$(NFS1\text{-}ISCU)_2$	47.7 +/- 0.0	145.7	136.4	6.89
$(NFS1\text{-}FDX2)_2$	48.3 +/- 0.1	141.5	139.4	3.99

Supplementary Table 3. Plasmids used in this study.

Plasmid	Relevant characteristics	Source
pACP-HA	yeast plasmid with yeast Acp1-HA	Gift of D.R. Winge [5]
p416	p416-MET25	[6]
pISDwt	*S.cer. ISD11* cloned into *BamHI/XhoI* of p416 expressing fused Isd11p-FLAG	This study
pISD1	*S.cer. ISD11 V12Q/L55Q variant cloned into BamHI/XhoI* of p416 expressing fused Isd11pV12Q/L55Q-FLAG	This study
pISD2	*S.cer. ISD11* T39D/A62N variant cloned into *BamHI/XhoI* of p416 expressing fused Isd11pT39D/A62N-FLAG	This study
pISD3	*S.cer. ISD11* Y34W/K38A/V68D variant cloned into *BamHI/XhoI* of p416 expressing fused Isd11pY34W/K38A/V68D-FLAG	This study
pISD4	*S.cer. ISD11* I75R/M78R variant cloned into *BamHI/XhoI* of p416 expressing fused Isd11p I75R/M78R-FLAG	This study
pISD5	*S.cer. ISD11* I75R/Y79R variant cloned into *BamHI/XhoI* of p416 expressing fused Isd11pI75R/Y79R-FLAG	This study
pISD6	*S.cer. ISD11* K17F/K21F variant cloned into *BamHI/XhoI* of p416 expressing fused Isd11pK17F/K21F-FLAG	This study
pZM2	Plasmid encoding *H. sapiens* mitochondrial NFS1 56-457 (Δ1-55)	Gift of S. Leimkühler [7]
pZM4	Plasmid encoding *H. sapiens* mitochondrial ISD11 1-91	Gift of S. Leimkühler [7]
pZM2Δtag	Plasmid encoding *H. sapiens* mitochondrial NFS1 56-457 (Δ1-55) without tag	This study
p24AC	Plasmid encoding *H. sapiens* ISCU1 protein 2-142	This study
p24ISCU_MI	Plasmid encoding *H. sapiens* ISCU1 protein 2-142 with M107I mutation	This study

Supplementary Table 4. DNA primer sequences used in this study.

Plasmid	Primers used for generation	Source
pZM2Δtag	Primer1: GATATACCATGGGCAGCAGCCTGCGACCTCTCTATATGG Primer2: CCA TAT AGA GAG GTC GCA GGC TGC TGC CCA TGG TAT ATC	pZM2
p24ISCU_MI	Primer1: CTG CAC TGC TCC ATC CTG GCT GAA GAT GC Primer2: GCA TCT TCA GCC AGG ATG GAG CAG TGC AG	p24AC

Supplementary References

1. Shi, R. et al. Structural basis for Fe-S cluster assembly and tRNA thiolation mediated by IscS protein-protein interactions. *PLoS biology* **8**, e1000354 (2010).
2. Marinoni, E.N. et al. (IscS-IscU)2 Complex Structures Provide Insights into Fe2S2 Biogenesis and Transfer. *Angewandte Chemie International Edition* **51**, 5439-5442 (2012).
3. Liu, J. et al. Structural characterization of an iron-sulfur cluster assembly protein IscU in a zinc-bound form. *Proteins* **59**, 875-81 (2005).
4. Roujeinikova, A. et al. Structural studies of fatty acyl-(acyl carrier protein) thioesters reveal a hydrophobic binding cavity that can expand to fit longer substrates. *J Mol Biol* **365**, 135-45 (2007).
5. Van Vranken, J.G. et al. The mitochondrial acyl carrier protein (ACP) coordinates mitochondrial fatty acid synthesis with iron sulfur cluster biogenesis. *eLife* **5**, e17828 (2016).
6. Mumberg, D., Müller, R. & Funk, M. Yeast vectors for the controlled expression of heterologous proteins in different genetic backgrounds. *Gene* **156**, 119-122 (1995).
7. Marelja, Z., Stocklein, W., Nimtz, M. & Leimkühler, S. A novel role for human Nfs1 in the cytoplasm: Nfs1 acts as a sulfur donor for MOCS3, a protein involved in molybdenum cofactor biosynthesis. *J Biol Chem* **283**, 25178-25185 (2008).
