# Supplementary material for: Structure and functional dynamics of the mitochondrial Fe/S cluster synthesis complex
Source: Nat Commun. 2017 Nov 3;8:1287. doi: 10.1038/s41467-017-01497-1 (PMC5668364; doi:10.1038/s41467-017-01497-1)
Supplement: Supplementary file 3 — Description of Additional Supplementary Files [file 41467_2017_1497_MOESM3_ESM.pdf]

Description of Supplementary Files

File name: Supplementary Movie 1
Description: Structural changes in the $(NIA)_2$ complex upon ISCU binding. NFS1, ISD11, ACP and ISCU are shown in orange, magenta, green and blue, respectively. Initial view shows the conformation of the $(NIA)_2$ complex found in the crystal. Upon ISCU binding (flying in from bottom left and top right) the $(NIAU)_2$ complex is formed and rigidifies by making a rotation around Pro71 of NFS1 (see Fig. 2a). This state represents the conformation found in the crystal of the $(NIAU)_2$ complex. Subsequent dissociation of ISCU relaxes the complex again and the $(NIA)_2$ proportion rotates back to the initial conformation.
